# 383. Assessing the 2021 IDSA Treatment Clostridioides difficile Guidelines in a Small, Community Hospital

**DOI:** 10.1093/ofid/ofac492.461

**Published:** 2022-12-15

**Authors:** Amanda Barner, Michael Maiullari, Shirley Yu, Shirley Yu, Mary Regan

**Affiliations:** Cambridge Health Alliance, Cambridge, Massachusetts; Cambridge Health Alliance, Cambridge, Massachusetts; Cambridge Health Alliance, Cambridge, Massachusetts; Cambridge Health Alliance, Cambridge, Massachusetts; Cambridge Health Alliance, Cambridge, Massachusetts

## Abstract

**Background:**

Cambridge Health Alliance (CHA) is an academic healthcare system in the greater Boston area that includes two acute care, community hospitals. Oral vancomycin has been the first line treatment option for initial *C. difficile* infection (CDI). In 2021, IDSA updated its guidelines to recommend fidaxomicin as the preferred treatment for initial episodes. At our institution, fidaxomicin is restricted to infectious diseases approval.The objective of this evaluation was to assess CDI recurrence rate and to identify a target population within our organization who may benefit from fidaxomicin as initial CDI treatment.

**Methods:**

A retrospective chart review of all inpatients ordered for oral vancomycin or fidaxomicin from January 2021 to February 2022 was conducted to assess treatment success, failure, and 60 day recurrence rate of CDI at two community teaching hospitals. Treatment success was defined as resolution of diarrhea or absence of diarrhea for two consecutive days after completion of therapy. Treatment failure was defined as continuation of symptoms for greater than 6 days after treatment initiation. Risk factors for recurrence evaluated were age > 65 years, immunocompromised status, fulminant infection, and use of vancomycin versus fidaxomicin.

**Results:**

A total of 47 patients administered oral vancomycin or fidaxomicin for CDI treatment between January 2021 and February 2022 were included in the analysis. Only one patient received fidaxomicin. The median age was 66 years and 55.3% female. Four patients were immunocompromised. Only one immunocompromised patient over 65 years had CDI recurrence. In our population, oral vancomycin had a treatment success rate of 85.1% and a recurrence rate of 2%. The one patient who received fidaxomicin did not have recurrence. Given the low recurrence rate, we were unable to identify significant risk factors associated with CDI recurrence in our population.

**Conclusion:**

In our population, oral vancomycin had a high treatment success rate, which correlates with current literature. Limitations of our analysis include its retrospective design, small sample size, and inclusion of few immunocompromised patients. Oral vancomycin remains the preferred treatment for an initial CDI episode in our population, as recurrence rate is low.

**Disclosures:**

**All Authors**: No reported disclosures.

